# Feasibility and Acceptability of Lee Silverman Voice Treatment in Progressive Ataxias

**DOI:** 10.1007/s12311-020-01153-3

**Published:** 2020-06-25

**Authors:** Anja Lowit, Aisling Egan, Marios Hadjivassiliou

**Affiliations:** 1 Speech and Language Therapy, School of Psychological Sciences and Health, Strathclyde University, 40 George St, Glasgow, G1 1QE UK; 2 Sheffield Teaching Hospitals NHS Trust, University of Sheffield, Sheffield, S10 2TG UK

**Keywords:** Progressive ataxia, Ataxic dysarthria, Voice quality, Speech therapy, Communication participation, Psychosocial wellbeing

## Abstract

Communication difficulties have considerable impact on people with progressive ataxia, yet there are currently no evidence-based treatments. LSVT LOUD® focuses on the production of healthy vocal loudness whilst also improving breath support, vocal quality, loudness and articulation in participating patients. This study aimed to investigate whether Lee Silverman Voice Treatment (LSVT LOUD®) can improve communication effectiveness in these patients. We performed a rater-blinded, single-arm study investigating LSVT LOUD® treatment in a population of patients with progressive ataxia including Friedreich’s ataxia (*n* = 18), spinocerebellar ataxia type 6 (*n* = 1), idiopathic cerebellar ataxia (*n* = 1), and spastic paraplegia 7 (*n* = 1). Twenty-one patients were recruited to the study, with 19 completing treatment. Sessions were administered via Skype in the LSVT-X format, meaning two sessions per week over a period of 8 weeks. Assessments included two baseline and two post-treatment measures and focused on outcome measures covering aspects ranging from physiological function to impact and participation. Results indicate improvements in patient-perceived outcomes for 14 of the 19 participants, in both speech and psychosocial domains. Speech data furthermore demonstrate significant improvements in prolonged vowel duration, and voice quality measures. Intelligibility and naturalness evaluations showed no change post-treatment. Patients reported high acceptability of the treatment itself, as well as administration by Skype. This is the largest treatment study for people with progressive ataxia published to date. It provides an indication that LSVT LOUD® can have a positive impact on communication in this patient group and could form the basis for larger-scale trials.

## Introduction

Ataxic dysarthria is a motor-speech disorder associated with cerebellar dysfunction which is prevalent in progressive ataxias. The characteristics of ataxic dysarthria include imprecise articulation, distorted vowels, voice changes, reduced speech rate, flat prosody and poor respiratory support [1]. These changes lead to reduced speech intelligibility and communication breakdown. In a recent survey by Ataxia UK [2], people with progressive ataxia identified speech and communication problems as one of the top three most troublesome symptoms of their disease with significant negative impact on their lives. Whilst our understanding of the nature of the communication problems experienced by these patients has improved significantly over time [3–13], a Cochrane Review on treatment efficacy for progressive ataxia syndromes concluded that “there is insufficient and low or very low quality evidence from either RCTs or observational studies to determine the effectiveness of any treatment for speech disorder” (Vogel et al., p. 1 [14]). Clinicians are therefore currently unsure of how to deal with ataxic dysarthria.

Vogel et al. [15] recently reported positive outcomes from a pilot study on speech treatment involving seven patients with autosomal recessive spastic ataxia of Charlevoix-Saguenay (ARSACS). Their treatment was home based, supported by an App that took participants through exercises addressing voice production and articulation. Another treatment approach that has already shown potential to increase communication efficiency across a range of motor speech disorders is Lee Silverman Voice Treatment (LSVT LOUD®, [16]). This intervention was originally designed to address the speech deficits associated with Parkinson’s disease (PD), in particular hypophonia, and thus focuses exclusively on increasing the healthy level of loudness in the patient’s speech. Its effectiveness has been demonstrated in a number of randomised controlled studies for PD [17–20]. In addition, there are a growing number of reports on its use in other disorders leading to dysarthria, such as cerebral palsy [21, 22], traumatic brain injury and stroke [23, 24], multiple sclerosis [25], and ataxic dysarthria due to thiamine deficiency [26]. Whilst most LSVT LOUD® studies have focused on loudness increases as their primary outcome measure, some also report positive effects on the wider articulatory system, such as improving breath support for speech, slowing down the rate, and improving the voice quality and articulation across these populations [18, 27, 28]. Although these reports often suffer from small sample sizes, and sometimes limitations in the breadth and rigour of their outcome measures, they provide an indication of the potential for LSVT LOUD® to achieve positive outcomes in patient populations other than PD and to impact on speech aspects beyond loudness.

The above research suggests that LSVT LOUD® could be a suitable intervention to treat people with progressive ataxia. However, there is a chance that the technique might not be suitable for all types of ataxic dysarthria, as the presence of concomitant problems such as fatigue, other health problems, and, in some ataxia types, impaired auditory processing or cognitive issues may limit its applicability. Furthermore, it is necessary to establish whether this approach is suitable to address the speech problems experienced by this group of patients, in particular with a view to the spasticity present in some individuals, for which LSVT LOUD® might be counterproductive. Assessments of the suitability of LSVT LOUD® for specific ataxia populations are therefore necessary. Consequently, the aim of our study was to perform a study into the effectiveness and acceptability of LSVT LOUD® to improve communication in people with dysarthria due to progressive ataxias.

One problem that has prevented large trials in this area before is the rare nature of the disorder. With the recent advances in telehealth technology, one way around this issue is to provide assessment and intervention remotely. Research evidence indicating the suitability for this management approach for acquired motor speech disorders is now relatively well established, for both assessment [29, 30] and treatment [31–36]. The studies furthermore report high patient satisfaction ratings. However, this research has mostly focused on patients with PD, and with predominantly mild motor difficulties. Issues of usability and acceptability are yet to be investigated for other populations such as people with progressive ataxia, and across a wider severity spectrum, to identify potential barriers that need to be considered.

Our research questions were as follows:

1. Does LSVT LOUD® result in positive changes to communication immediately and 2-month post-treatment in speakers with progressive ataxia and dysarthria?
2. Does LSVT LOUD® lead to any undesirable outcomes such as increased fatigue level, or impact on voice quality?
3. What is the patient’s experience of LSVT LOUD® delivered by Skype as a treatment regime?

The study is reported according to CONSORT 2010 statement: extension to randomised pilot and feasibility trials [37].

## Materials and Methods

### Trial Design

This 2-year study was a rater-blinded, single cohort design of patients with dysarthria due to progressive ataxia, using a single-study arm—LSVT LOUD® treatment. Eligibility criteria were adjusted in two ways in order to facilitate recruitment within the given time frame of 15 months. First, we extended inclusion criteria to speakers with severe dysarthria providing they successfully completed the stimulability assessment for the intervention approach. Second, the study was initially restricted to speakers with Friedreich’s ataxia as per funder focus. However, the study was later opened up to other types of progressive ataxias, resulting in the inclusion of 3 patients with other forms of this ataxia.

### Sample Size

The study was intended to function as a feasibility study for a larger RCT on the one hand, and already contribute credible evidence towards treatment of dysarthria in progressive ataxia on the other. No previous research was available on this population to enable the calculation of an appropriate sample size. A sample size of 20 was chosen as this was deemed feasible within the available timescale of 15 months and, in addition, aligned with the recommendation of patient numbers for studies following on from single case reports [38]. This permitted us to already contribute the results of this study to the evidence base for treatment of ataxic dysarthria. The treating speech and language therapist (SLT) continuously monitored each patient for adverse reactions to treatment, in order to allow for necessary adjustments to be made to the intervention if necessary. None were reported.

### Participants

Eligibility criteria for the study included a confirmed diagnosis of progressive ataxia, the presence of ataxic dysarthria, the absence of a functional voice disorder other than can be expected as part of the ataxia, age above 16 years, ability to follow the assessment and treatment tasks, and availability of technology to complete assessment and treatment tasks via Skype.

Advertising took place via the funder website and social media campaigns, as well as information leaflets posted in a specialist ataxia clinic at the Sheffield Ataxia Centre, Sheffield Teaching Hospitals NHS Trust, and University of Sheffield. As the study was funded jointly by charities in the UK and Switzerland, recruitment took place in both locations. All participants self-selected and contacted the research team to discover more information about the study. Participants were provided with study information by email. Suitability to participate was established during a phone call and participants subsequently returned consent forms back to the study team.

### Assessment and Treatment

The study included four assessment points, including multiple baseline assessments (two sessions administered 2 weeks apart prior to treatment), and two post-therapy assessments, one within 1 week of completing treatment, and another 8 weeks post-treatment. Assessments were conducted by the first author who was not involved in the treatment of participants.

LSVT LOUD® is an intensive treatment that consists of four 60 min sessions per week over the course of 4 weeks. In addition, home practice is required: 10 min once a day on treatment days and twice a day on non-treatment days [17]. However, an extended version (LSVT-X), consisting of 2 sessions a week over 8 weeks, has been shown to result in comparable speech outcomes [39]. Following consultation with a focus group of four people with progressive ataxia, it was decided to offer participants LSVT-X due to concerns about impact on fatigue levels of the more intense treatment. Sessions generally lasted between 50 and 60 min and followed the prescribed treatment schedule and tasks, and participants were advised to follow the suggested home-practice schedule [39]. As indicated above, LSVT LOUD® focuses on establishing a healthy loud voice, which is often lacking in speakers with PD. Whilst hypophonia has also been reported in people with ataxia, we anticipated that this would not be the case for all study participants. Treatment thus varied depending on the needs of the individual, with a focus on a healthy, unforced voice production for all speakers, and emphasis on a louder voice only for those with symptoms of hypophonia. As part of the aim of this feasibility study was to establish that LSVT would not be harmful to participants, their voice quality and other speech characteristics were carefully monitored throughout the treatment.

Sessions were administered by two SLTs, the first treated participants 1–3, the second the remaining participants. Both were experienced, LSVT LOUD®-trained clinicians, who had treated the minimum recommended number of patients before becoming involved in the treatment study.

Given the distance of study participants’ homes to the investigators, both assessment and treatment sessions were delivered remotely. In consideration of cost-effectiveness issues for health services, we did not purchase any tailored software such as the LSVT LOUD® companion, but instead, used off-the-shelf, freely available tools for communication (Skype version 8.48.0.51) and to record assessment sessions (Audacity® version 2.2.2). In addition, participants were supplied with a low-cost loudness meter (Grandbeing Schallpegelmesser). They were given access to the university’s cloud server to securely upload their assessment recordings after the session. In addition, Skype calls were audio recorded with their permission as a backup during assessment sessions using the CallNote App.

### Assessment Tasks

Both quantitative and qualitative data were collected from participants to capture as many therapy outcomes as possible. These covered both speech (assessed in all four sessions) and psychosocial impact, communication participation, and fatigue measures (collected in assessment session 1 and 3). In addition, demographic data and medical history were collected from participants in session 1.

Speech assessment included tasks to assess both individual speech components and more natural connected speech. Tasks were administered in semi-randomised order, structured tasks were always presented first and in the same order, speech tasks were presented second, but in randomised order.

Data presented in this paper relates to the following tasks:

1. vowel prolongation, best of 3 attempts,
2. a reading passage (The Caterpillar [40])
3. a 1-min monologue about a topic of choice (e.g. a holiday, hobby, or recent memorable event).

Fatigue was measured using the Fatigue Impact Scale (FIS) [41] to assess whether participation in treatment had adversely affected the participants’ fatigue levels.

To evaluate self-perception and impact of dysarthria pre-therapy, we interviewed participants using the standard pre-treatment questionnaire of the LSVT LOUD® programme, and also asked them to complete the Voice Handicap Index (VHI) [42], and the short form of the Communication Participation Item Bank (CPIB) [43]. The same questionnaires were used immediately post-treatment (session 3), in addition to a further interview where we discussed changes after treatment as well as experience of the treatment process in relation to schedule, content, and administration by Skype.

### Analysis

This paper focuses on the primary outcomes measures of maximum phonation time and voice quality in vowel prolongation, and intelligibility and naturalness of connected speech. Loudness level, which is another frequent outcome measures in LSVT studies, could not be reported as results were too unreliable due to participants recording themselves remotely. Participants had been asked to take several loudness meter measurements during the session, which were later compared with the decibel values from the recording. It emerged very quickly that the relationship between actual decibel level as indicated on the loudness meter and those of the recordings were not constant. In addition, some participants were observed to be shifting position considerably during the assessment sessions, thus changing the distance to their microphones. The resulting variations in loudness measures reached as much as 3 dB, which was deemed too great to be able to confidently attribute changes in loudness to treatment outcomes or estimate the average increase in loudness achievable through LSVT LOUD®. This measure was therefore excluded from the evaluation.

Secondary outcomes, i.e. measures of psychosocial impact, fatigue ratings and patient perceptions are also reported. All examiners were blinded to the time-point of the samples they analysed.

#### Vowel Prolongation

The vowel prolongation task was the basis for maximum phonation time (vowel length) and voice quality measures. Vowel length in milliseconds was determined from oscillographic and wide-band spectrogram data in Praat ([44], version 6.0.43). In addition, the data were evaluated perceptually by four experienced SLTs using the GRBAS [45]. This tool provides scores for Grade (G—overall severity), roughness (R), breathiness (B), asthenia (A—weak voice), and strain (S). Listener inter- and intra-rater agreement was very good with Cronbach’s alpha levels of .836 and .815 respectively.

#### Reading

The reading task was evaluated for intelligibility and naturalness. To avoid effects of familiarization with the speech material, listeners were asked to score reading samples using direct magnitude estimation (DME). This method uses a standard, which is given a score of 100, and asks listeners to rate a given speech sample in relation to this standard, where a score of 50 represents a sample half as intelligible or natural, and a score of 200 twice as intelligible or natural as the standard. The standard was a speaker with moderate dysarthria who was not included in the study (participant 12). Samples were presented in groups of 5, i.e. listeners heard the standard, followed by the recordings of the four assessment sessions from each participant in randomised order. The reading samples consisted of an excerpt from the middle of the reading passage of approximately 30 s length. Listeners were instructed to listen to the whole sample before scoring to account for potential variations in speech quality. To arrive at an overall score per sample, the geometric mean was calculated. The listeners consisted of four highly experienced SLTs familiar with neurodegenerative disorders different to those who had evaluated the voice samples. Agreement between listeners for the DME scores for assessment 1 was high at 0.877 for intelligibility and 0.833 for naturalness ratings.

#### Monologue

One participant joined the study from Switzerland; she was sufficiently fluent in English to perform the assessment tasks and follow treatment instructions. However, she was asked to use her native French in order to collect a more representative sample of her natural speech performance. To accommodate this fact, the monologue data were judged by three naïve English-French bilingual listeners. The listeners scored samples of approximately 30 s length from the middle of the monologue on a 9-point scale that accounts for intelligibility as well as listener effort [46]. The scores were used to determine dysarthria severity (Table 1) as well as post-treatment effects. All naïve listeners had appropriate hearing ability and no prior experience of ataxic or other types of disordered speech. They were blinded as to assessment session. Cronbach’s alpha for listener agreement for the monologue evaluation of assessment 1 was .939, which indicates high levels of agreement.

**Table 1:** Participant details

Participant	Age	Gender	Diagnosis	Years since diagnosis	Motor impairment	Intelligibility deficit in monologue (0–9 scale)
1	45	M	FRDA	17	moderate	7.5 mild
2	32	F	FRDA	22	severe	2 severe
3	36	F	FRDA	25	severe	2 severe
4	52	M	FRDA	14	moderate	6.5 mild - moderate
5	40	M	FRDA	24	severe	NA
6	59	F	FRDA	46	moderate	4 moderate
7	23	F	FRDA	13	moderate	7.5 mild
8	54	F	FRDA	10	moderate	8.5 normal
9	75	F	FRDA	17	severe	7.5 mild
10	31	F	FRDA	22	moderate	7 mild - moderate
11	40	M	FRDA	21	severe	9 normal
12	32	M	FRDA	21	severe	5.5 moderate
13	25	M	FRDA	10	moderate	3.5 moderate - severe
14	48	F	FRDA	30	moderate	5 moderate
15	29	M	FRDA	21	severe	5.5 moderate
16	19	M	FRDA	9	moderate	5 moderate
17	31	F	FRDA	22	severe	4.5 moderate
18	71	F	FRDA	12	moderate	7 mild moderate
19	49	M	SPG7	5	moderate	5.5 moderate
20	70	M	ICA	12	moderate	6.5 mild - moderate
21	73	M	SCA6	19	moderate	4 moderate
*Summary:*	Mean: 44.5 SD: 17.3	M: *n* = 11 F: *n* = 10		Mean: 18.7 SD: 8.9	Moderate *n* = 13 Severe *n* = 8	Mean: 5.5 SD: 2.1

*M* male, *F* female; *FRDA* Friedreich’s Ataxia, *SCA6* Spino-Cerebellar Ataxia Type 6, *ICA* Idiopathic Cerebella Ataxia; *SPG7* Spastic Paraplegia 7

### Statistical Analysis

Not all data were distributed normally, and non-parametric statistics were therefore used throughout, using the Friedman Test to look for changes across time, and the Wilcoxon signed-rank test for the post hoc analyses or in cases of paired comparisons (some vowel prolongation and monologue data). Bonferroni corrections were applied in cases of multiple comparisons. Where correlations were calculated between participant characteristics and post-treatment change, the latter represented the percentage difference between the mean of assessments 1 and 2, and assessment 3. Listener agreement was calculated with the Inter-class correlation coefficient as more than two listeners were involved in each exercise.

## Results

### Recruitment

The recruitment period lasted 15 months. During this time, one participant was recruited through the NHS, the rest through charity advertising and information sharing on social media sharing by previous participants.

At completion, the study included 18 patients with FRDA, one with Spastic Paraplegia 7 (SPG7), one with Spino-Cerebellar Ataxia Type 6 (SCA6), and one with idiopathic cerebellar ataxia (ICA). A further seven people contacted the study team about participation. Five of these had ataxias other than FRDA and were added to a waiting list in case the study opened up to these types of ataxia later. Two of those joined the study at a later date. One person with FRDA established contact but chose not to participate due to work pressures, and a further person with FRDA contacted us too late to be included in the trial.

### Baseline Data

Table 1 provides details of patients recruited to the study, including medical history and dysarthria features. As the majority of participants lived a considerable distance from the consulting neurologist and were not due to a routine appointment during the study duration, no up-to-date neurological examination could be conducted as part of this study. Instead, we applied a rough grading of their motor ability as mild (can walk unaided), moderate (needs walking aids), and severe (wheelchair bound). Considering the fact that the feasibility assessment focused on the appropriateness of the speech treatment approach and administration of this via Skype, this was deemed appropriate for the purpose of this study. Table 1 shows that the majority of our participants were rated as showing moderate or severe motor impairment. On the other hand, most had a mild-to-moderate level of speech impairment, with only a few located at the lower moderate to severe end of the spectrum.

### Adherence

Of the 21 patients recruited, 20 commenced and 19 completed treatment (Table 2). One speaker with FRDA provided consent, but then became too unwell to participate and no assessment data were collected from him. One other participant (participant 12) commenced treatment which was put on hold after 6 sessions due to suspicion of vocal pathology. This only became apparent after his speech performance began to improve as a result of treatment, and he was able to produce a prolonged sound long and loud enough to highlight potential problems with his voice. The participant was advised to seek ENT examination to ensure speech treatment would not adversely affect his vocal health. The resulting delay meant that he could not rejoin treatment and he was therefore categorised as a non-completion. A further participant became hoarse during the second assessment. It was initially assumed that this was due to a cold and treatment was started, but the problem persisted. He was again advised to seek medical examination and rejoined the study at a later date.

**Table 2:** Adherence data, indicating number of sessions attended and number of interruptions (target number of sessions—16)

Participant	No. of sessions administered	No. of sessions rescheduled	No. of interruptions in treatment > 1 session
1	16		
2	16	2	
3	16		1
4	15	1	1
5	No treatment		
6	16		1
7	16		
8	14	2	2
9	16	1	
10	16		1
11	13		
12	5—discontinued		
13	16		
14	13		1
15	14		
16	16		1
17	16		
18	16		2
19	16		1
20	14		
21	15	1	

All but four of the participants experienced gaps in the treatment regime due to ill health or holidays. This could take the form of single sessions within a week or interruptions of 1 week or longer. When individual sessions were missed, it was attempted to reschedule them, but this was not possible in all cases. Twelve of the 19 participants completing treatment received the full number of sessions, two participants missed 1 session, three participants 2 sessions, and two participants 3 sessions. Longer interruptions tended to last for 1 to 2 weeks, but extended to 4 weeks in one case.

### Numbers Analysed

Overall, most data are complete across tasks and measures. VHI questionnaire data are missing from 3 participants due to a clerical error. One participant had visual problems and was therefore unable to complete the reading assessment.

### Outcomes

#### Prolonged Vowel Measures

Maximum phonation time shows a significant change over time (Friedman test: *p* = .003, df = 3). Bonferroni corrections were applied to the post hoc tests in relation to two hypotheses; no change between assessments 1 and 2, and 3 and 4 (2 comparisons, *p* < .025), and change between pre- and post-treatment assessments (4 comparisons, *p* < 0.0125). The post hoc tests indicate no significant difference between the two pre-treatment (*p* = .116) or post-treatment sessions (*p* = .081). On the other hand, comparison between pre- and post-treatment sessions shows significant differences between the session 2 and both post-treatment assessments (session 2–3: *p* = .001; session 2–4: *p* = .001), whereas comparisons between sessions 1 and 3 and 4 were not significant (session 1–3: *p* = 0.014; session 1–4: *p* = .131). Table 3 provides the group means and standard deviations for each assessment point. As suggested by the high standard deviation, performance varied considerably between participants, with the poorest performer only achieving a length of 2.7 s in session 1, and the highest performer 23.6 s, with four participants performing within the normal range ([47]). The range of improvements varied as well. A comparison of the mean of assessments 1 and 2 with assessment 3 indicates that 13 participants improved more than 20%, the largest change showing an increase from 2.7 to 16.2 s. At the same time, six speakers changed their vowel length by less than 5% or performed slightly worse. These included the four speakers who already performed within the normal range, as well as one participant with a pre-existing lung condition that limited his performance. If those five speakers are excluded from the data set the pre- to post-treatment comparisons all become significant (session 1–3: *p* = .002; session 1–4: *p* = .001; session 2–3: *p* = .002; session 2–4: *p* = .003).

**Table 3:** Prolonged vowel data: vowel length and GRBAS scores

Session	1	2	3	4
Length	9.80 (6.56)	8.92 (6.30)	12.78 (5.98)	11.62 (4.88)
G	1.95 (0.52)	1.74 (0.60)	1.61 (0.63)	1.33 (0.57)
R	1.12 (0.74)	1.09 (0.61)	0.70 (0.56)	0.91 (0.52)
B	0.83 (0.56)	0.88 (0.59)	0.58 (0.35)	0.42 (0.46)
A	0.96 (0.72)	0.96 (0.56)	0.45 (0.38)	0.45 (0.41)
S	1.37 (0.75)	1.24 (0.67)	1.34 (0.74)	0.93 (0.55)

Values denote means and (standard deviations) for vowel length (in ms) and GRBAS scores (0–5 scale)

*G* grade, *R* roughness, *B* breathiness, *A* asthenia, *S* strain

In summary, of the 13 participants who were expected to improve in their maximum phonation time, twelve achieved this. Furthermore, participant 12, who had his treatment terminated early and is thus not included in this analysis, also demonstrated noticeable improvements in maximum phonation time within the first week of treatment. The data thus suggests that LSVT® was generally successful in improving participants’ breath support for speech where this was reduced. Non-FRDA participants performed well within the range of the remaining speakers in relation to their baseline performance and degree of change after treatment, thus not suggesting any influence of genotype on maximum phonation time.

The second measure taken from the vowel prolongation task was perceptual voice quality as reflected by the GRBAS evaluation (Table 3). Although the scores were relatively mild across the group, none of the participants were scored by all listeners as having a value of 0 (no impairment) across any of the dimensions pre-treatment. The highest score awarded was 3, indicating at most a moderate impairment of voice quality. For the statistical analysis, Bonferroni corrections were set as *p* < .010 for the Friedman test (5 comparisons), and as specified for maximum phonation time above for the post hoc tests. The Friedman test results indicate significant change across all variables but Strain (Table 4). Post hoc tests show significant differences from at least one of the pre-treatment sessions to post-treatment, with no significant change between the two pre-treatment or post-treatment sessions. The change over time for Grade, Roughness, Breathiness, and Asthenia can thus be attributed to a treatment effect. As an indication of the range of performance, the comparison of the mean values of assessment 1 and 2 with assessment 3 showed improvements in overall voice quality (Grade) for eight participants, in Roughness for 15, Breathiness for 14 and Asthenia for 16 speakers. The remaining participants showed no or small negative change. Patterns for Strain were more variable, with only eight participants showing improvement, five having no or minimal change, and a further four showing some more noticeable deterioration, resulting in the statistically not significant result. Comments from listeners suggest that this might have been due to these speakers forcing their voice to some degree towards the end of the prolonged vowel to extend their duration as much as possible.

**Table 4:** Statistical results for pre- and post-treatment comparisons

	Friedman	Pre-treatment	Pre- to immediately post-treatment		Pre- to 8 weeks post-treatment		Post-treatment
Session:		1–2	1–3	2–3	1–4	2–4	3–4
Length	.003	.116	.014	*.001*	.131	.011	.081
G	< 0.001	.499	*.002*	.056	*< .001*	*.001*	.043
R	0.009	.673	*.006*	*.008*	.108	.078	.028
B	< 0.001	.839	.048	.028	*< .001*	*.001*	.140
A	< 0.001	.499	*.002*	.056	*< .001*	*.001*	.043
S	0.466	---	---	---	---	---	---

All values denote *p* values. Significant results are marked in italics

*G* grade, *R* roughness, *B* breathiness, *A* asthenia, *S* strain

**Fig. 1:**
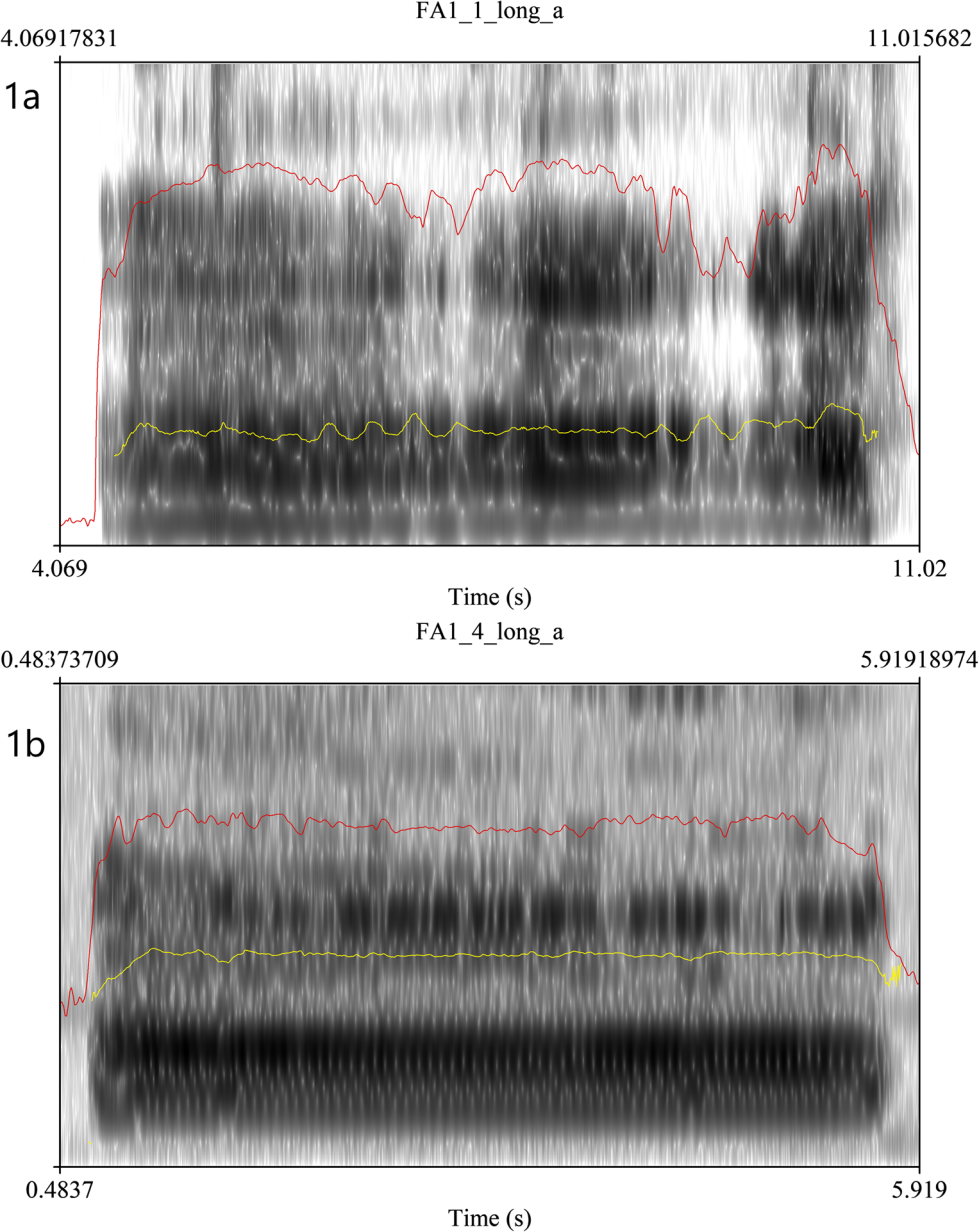
Oscillogram and spectrogram plots of prolonged vowel for session 1 (**a**) and session 4 (**b**) for participant 1. The red line represents the loudness contour, and the yellow line the pitch contour

Figure 1 provides a visual example of some of the positive changes perceived by the listeners. Figure 1a (participant 1, pre-treatment) shows an unsteady pitch, large variations in loudness, and some aperiodicity of phonation, resulting in a perception of roughness. Figure 1b (participant 1, post-treatment), on the other hand, demonstrates a smoother, more periodic vowel phonation with steady pitch and loudness throughout, reflecting better control of the vocal mechanism. There was again no evidence in an influence of genotype on voice quality, and the range of scores and degree of change from pre- to post-treatment was comparable across participants with FRDA and other types of ataxia.

Whilst there was no evidence of influence of genotype on the above measures, a correlational analysis demonstrated a significant relationship between severity (as measured by monologue intelligibility) and maximum phonation time (*r* = .646, *p* = .003), as well as between maximum phonation time and voice quality at assessment 1 as reflected by the Grade score (*r* = − .540, *p* = .017). However, there was no significant correlation between severity and the degree of change in maximum phonation time (*r* = − .276, *p* = .253) or Grade (*r* = .067, *p* = .785) after treatment (percentage change from assessment 1 to 3), or between maximum phonation and the change in Grade (*r* = .270, *p* = .263). The data thus show that participants with milder levels of dysarthria had longer maximum phonation time and better voice quality. However, baseline severity did not necessarily predict to what degree those measures would improve.

#### Intelligibility and Naturalness

**Fig. 2:**
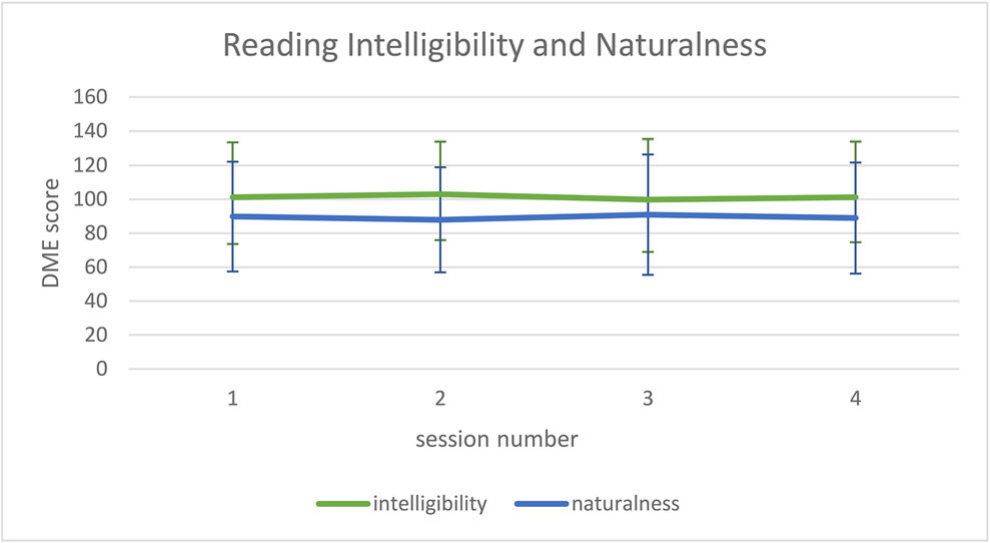
Intelligibility and naturalness DME ratings (mean and SD) for the reading samples across all four assessment sessions

Figure 2 shows the results for intelligibility and naturalness in the reading passage for expert ratings across all four time points. The Friedman test across did not indicate any significant changes over time in intelligibility or naturalness (*p* = .813 and *p* = .989 respectively). A similar lack of change in intelligibility was identified by the naïve listeners in the monologue (*p* = .333). Qualitative inspection of these data reveals relatively small deviations from baseline, indicating that this was due to little change being perceived rather than change occurring in different directions. This applied equally to all participants, with no effects of genotype noticeable. There was also no significant correlation between severity and extent of change in intelligibility in reading (*r* = .059, *p* = .816) or the monologue (*r* = −.006, *p* = .980), i.e. the degree or direction of change did not depend on the baseline intelligibility level of the speaker.

#### Psychosocial Outcomes and Participant Perceptions

None of the questionnaires showed any significant differences between pre and post-treatment sessions (CBIP: *p* = .154, VHI: *p* = .056, VAS: *p* = .778). On the other hand, post-treatment interviews indicated that the majority of participants felt their communication had improved after treatment. Most participants indicated noticeable improvements in at least two speech or psychological dimensions; however, two patients only described minimal changes (participants 4 and 9) and two further patients reported no changes post-treatment (participants 10 and 14). The most common dimensions highlighted by those who reported treatment effects were improved loudness and/or better control over their voice (14/17 patients)—*“Because of the changes in my voice I used to sound anxious in meetings, but now that I have the strategies my presentation went really well”; “It’s helped me to control my voice”*; clearer speech and/or less need to repeat (13/17 patients), and being able to speak in longer phrases, for a longer time or both (13/17 patients)—*“Before, it took a lot more effort to pronounce words”*; *“I find it easier to complete all the syllables now”*; and *“Before, I’d say “I can’t be bothered”, but now I’ve been able to be more involved in conversation”*. In about 50% of these cases, these reports were corroborated by friends and family who in some cases were unaware of the fact that the participant had undergone treatment recently—*“I recently met with some college friends who I’d not seen for 2 years and they commented I sounded better than last time”*. An interesting finding in relation to loudness was that some participants initially reported worries about sounding aggressive when speaking at a normal volume, and consciously reduced their speaking volume. Rather than effecting physiological change to improve hypophonia, the treatment addressed a psychological dimension in these speakers. A further psychological outcome that was frequently highlighted was an increase in confidence or reduction in anxiety, in many cases leading to increased communication participation—*“I’m not worried that people will ask me to repeat anymore”* and *“I used to avoid phone conversations but I’m fairly confident now”*. Despite the lack of significant changes in the questionnaire data, participant responses thus indicated positive outcomes after treatment for both speech and psychosocial aspects. As demonstrated in Table 5, there was no noticeable difference in terms of reported outcomes for the non-FRDA participants (speakers 19, 20, and 21).

**Table 5:** Patient perceptions of changes in communication and psychosocial dimensions post-therapy

Participant	Louder	Clearer	Longer phrases/speaking time	Better pacing/breath management	Better pitch/loudness control	Corroboration by others	Increased confidence/ reduced anxiety
1	1	1					1
2	1	1	1	1		1	1
3	1	1	1			1	1
4	1	1	1				
6		1	1	1			1
7	1		1				
8	1	1			1	1	1
9		1	1				
10							
11	1	1	1	1			1
13	1	1	1	1		1	
14							
15	1		1			1	
16	1	1			1	1	1
17	1		1				
18	1	1	1			1	1
19	1	1	1				
20		1	1				1
21	1						1
Total	14	13	13	4	2	7	10

#### Acceptability of LSVT LOUD® and Skype Delivery

The post-treatment interview also questioned participants about their experiences of the treatment programme and the remote provision using Skype. All respondents indicated that the treatment had been relevant and addressed the areas of speech impairment that they were concerned about. Two participants indicated they would have liked to work more on articulation. All were able to cope well with the two sessions per week regime, there was variable response in relation to whether they would have managed the usual four sessions a week as treatment was tiring. There was also a wide variation in terms of home practice adherence, with some participants indicating practising the recommended 4–5 times a week, and others only managing once or twice in addition to their therapy sessions.

All but one participant indicated that they preferred remote treatment to face-to-face sessions. The main reasons provided were reduced fatigue from not having to travel to clinic, greater flexibility to fit sessions around other activities and/or reduced travel time, particularly for those still in employment. None of the respondents felt that patient–therapist relationships had been impacted by the remote treatment, or that technical problems affected treatment provision. They did indicate though that the remote assessment had been more of a problem in terms of dealing with the technology to record their speech and upload the data.

### Harms

None of the participants reported any harm as a result of participating in this trial, and there was no evidence of negative sequelae from observations during intervention or highlighted by the subsequent data analysis either. In particular, the hypothesis that voice quality might be adversely affected by the treatment was rejected by our analysis.

All participants had reported issues with fatigue in the initial interview, which was frequently mentioned as one of the three most prominent issues affecting their lives. The mean overall score (VAFS) on the Fatigue Impact Scale (FIS) was *x* = 5.17 (SD = 2.4, range 3–10 where 0 is worst and 10 is normal). Scores for the nine individual FIS categories did not change significantly from pre- to post-therapy (*p* = .251). There was therefore no indication that treatment had adversely affected the participants’ fatigue levels. Patient reports in the post-treatment interview confirmed this fact, although a number of participants reported that the treatment had been strenuous.

## Discussion

This feasibility study presents the outcomes of LSVT LOUD® treatment for patients with progressive ataxia. Overall, study outcomes were positive for measures related to voice quality and breath support for speech as measured by vowel prolongation. Formal measures of intelligibility or psychosocial impact did not show any statistically significant changes post-intervention. However, patient reports in interviews suggested beneficial effects on both dimensions for the majority of participants. Furthermore, participants reported that LSVT LOUD® was an appropriate and acceptable intervention approach, and particularly liked the remote administration via Skype. Neither formal measures nor patient reports indicated any negative impact on fatigue levels with the current LSVT-X treatment regime.

There is only one other study to date that has investigated the effects of speech treatment in a group of seven speakers with progressive ataxia [15]. This study’s approach included a wider range of treatment targets, including voice and loudness production, as well as articulatory practice. The authors report positive outcomes for intelligibility and naturalness, but not for vocal control, although this study did not go into the same amount of detail of analysis as the current investigation. The only other comparable study is the single case report of non-progressive ataxia [26]. Again, our intelligibility outcomes do not match those reported for this case; however, they compare favourably with the vowel prolongation measures and self-reported psychosocial benefits.

Whilst the GRBAS ratings for our participant group suggested mostly milder levels of dysphonia in line with the literature, improving voice quality and vocal stability should still be a consideration in treatment planning ([9]). In this regard, our results on improved voice quality were positive. One of the reservations of using LSVT LOUD® with this patient group had been a possibly contraindication of the effortful therapeutic approach in the presence of spasticity, which can be a feature in FRDA. However, our results suggest that not only did LSVT LOUD® not cause any harm in this respect, it actually had beneficial consequences for voice quality. In line with previous reports on other dysarthria types (e.g. [24, 31]), LSVT LOUD® thus represents a viable option to improve vocal stability in ataxia. Participant comments furthermore suggested wider benefits to communication as a result of the noted improvements in vocal quality such as increased communication participation due to the reduced effort required for communication or the increased control over their voice.

The results on improved breath support as reflected in increased vowel prolongation were furthermore encouraging. Whilst we would have liked to capture the impact of this in connected speech, this was not possible, as qualitative inspection of the data indicated that participants both increased and reduced their phrase lengths after treatment. The latter was not due to any negative impact of treatment, but simply the fact that they had learnt to manage their breath support better by placing more pauses in strategic places. This, however, meant that phrase length was not an appropriate outcome measure to use in this study. A number of participants commented positively on this feature during interview though, and more importantly, spoke about the ability to take part in conversation for longer.

The lack of improvement in intelligibility and naturalness scores was unexpected, but there are several possible explanations for this, the first being issues with adherence. Our participants experienced a high number of interruptions and gaps in their treatment course (Table 2), which could have affected their outcomes. On the other hand, whilst Vogel et al. [15] report a 100% completion rate of their protocol, no information is provided about interruptions. Sapir et al. [26] do not mention adherence being an issue in their case report. In addition, Vogel et al. [15] used a home practice App which ensured regular practice by participants. This was not monitored formally in our study, but judging from patient reports, the suggested regime was not always maintained. Finally, Vogel et al.’s [15] participant group was of lower severity level than included in this study. Whilst we could not identify any relationship between level of speech impairment and post-treatment change in our study, the lower level of disability could have further contributed to greater adherence and thus possibly better outcomes in their study.

Other factors include influences of recording quality on listener ratings, or the fact that participants might not have been feeling well at the time of the post-treatment assessment, which was reported by some individuals. In addition, many participants struggled with the recording procedure and could thus have been distracted from the speech tasks during assessment, and as a result might not have used the strategies practiced in treatment during the assessment sessions. The fact that other people commented on improvements and participants reported a reduced need to repeat themselves could indicate that they used their strategies in real-life situations when they needed to make themselves understood, but not during the rather artificial assessment context. Finally, it should be noted that intelligibility improvement in PD as a result of LSVT LOUD® is often associated with the resulting increase in loudness. As the current participant sample did not demonstrate significant levels of hypophonia at baseline on the whole, this factor was not a contributor here. Instead, we hypothesised that the additional effort associated with loud voice production might have resulted in improved intelligibility, but this was not the case. It is thus possible that the intelligibility deficits caused by the poor coordination of movement associated with ataxic dysarthria is less likely to respond to increased effort in speech production than the reduced range or speed of movement reported in PD (e.g. [31]) or non-progressive dysarthria ([23, 27]).

A further unexpected result was the lack of improvement for the scores of the formal questionnaires on impact and participation in view of the positive reports on treatment outcomes emerging from the patient interviews. Again, there are potential explanations for this finding. First, the scoring of these questionnaires might have been insufficiently sophisticated to capture the perceived change. For example, the CPIB uses four categories ranging from “not at all” to “a little”, “quite a bit” and “very much”, which might not have picked up some smaller improvements. Second, the questions might not necessarily have picked up on the dimensions that improved in participants, e.g. the amount of interference of their dysarthria in talking to people they do not know or talking on the phone might not have changed, but they reported feeling more confident about it, which is not captured by the questionnaire. Finally, the participants did not have their initial responses available when scoring the post-treatment questionnaires and a change of awareness or perception of certain features as a result of the treatment process could thus have led to scoring an item as being worse on the questionnaire when the participant actually expressed the opinion that this had improved in their interview. An important point to remember is that these questionnaires were not designed as outcome measures but as status questionnaires, intended to highlight to the clinician what impact the speech or voice disorder is having on the patient. The current study suggests that their use as outcome measures needs to be considered carefully and ideally be supplemented with qualitative interview data from participants.

## Limitations and Interpretation

There were some limitations to our paradigm that might have impacted on our results and/or should be addressed in future larger trials. First, running this study under typical health service provider conditions without any specialised software has impacted on the fidelity of the outcome measures. This relates in particular to the quality of the speech recordings and the accuracy of the loudness measures. Although there was sufficient good-quality material available for each participant to capture our outcome measures, part of the data had to be disregarded at times as the quality was too low. Whilst the loudness readings taken by the participants were sufficiently accurate for treatment purposes, they fluctuated too much by e.g. speakers shifting position and thus distance to the microphone to yield reliable data necessary for the outcome measurement. We suggest that any future study at least supplies participants with lapel or headmounted, calibrated microphones to ensure good quality and accurate data collection or, preferably, performs assessment face to face, as participants reported this part of the study as taxing.

Another methodological issue that should be addressed in a future study is the way the qualitative data are collected. The current interviews did not refer to the questionnaire data, as not all participants returned this information in time for the first post-treatment assessment session. It was thus not possible to explore potential discrepancies between the interview and questionnaire data. One reason why interview reports were more positive than formal ratings could of course be that participants felt under some pressure to report positive outcomes as they were interviewed by one of the study investigators (though not the person who provided the treatment). However, whilst this might explain some of the differences, we feel that the interview data are sufficiently true to be used as an outcome as (1) participants also reported negative issues or the fact that nothing had changed, and (2) reports of what had changed in their communication were sufficiently consistent across participants to suggest that these were real changes experienced, as no prompts were given as to what aspects might have altered in their speech. As indicated above, we therefore believe that the issue lay with the questionnaires rather than interview data, and that they should be cross-checked with patient interviews.

The final point that should be considered in a future trial is the fact that the choice of LSVT-X (8-week period) possibly led to more cancellations due to participants feeling unwell, going on holiday or having to pursue other appointments than is usually reported for traditional LSVT LOUD® (4-week period). Whilst there were mixed reports from participants on whether they felt they could have dealt with the more intensive programme, this option should not be disregarded in future considerations.

On the other hand, treatment delivery by Skype was fully supported by all participants. Only one person expressed a preference for face-to-face therapy, although they still felt that the Skype sessions provided the same level of therapeutic benefit. Others expressed that they could not have participated in treatment had it not been delivered remotely, particularly those who were still in employment and would not have been able to fit the hours into their work schedule, and those suffering from severe fatigue issues.

## Conclusion

Our study represents the largest clinical trial conducted on people with progressive ataxia to date. Post-treatment comparisons indicate improvements in physiological functioning (voice quality, breath support), as well as speech production, communication participation, and some psychological dimensions (confidence, anxiety) as perceived by the participants, in both speakers with FRDA and other types of progressive ataxia. Whilst the latter could potentially be attributed to a placebo effect, the physiological changes and some of the reported speech outcomes are more likely to be a result of speech intervention, thus indicating positive treatment effects. Intelligibility and naturalness as rated by unfamiliar listeners did not change significantly. However, whilst increased intelligibility is often regarded as an important indicator of improved communication, our patient-reported outcomes suggest that this can also be reflected by other measures, such as lessened anxiety whilst communicating, or reduced effort required for speaking.

In the current climate where many health professionals are unsure about how best to support patients with progressive ataxia, we would therefore argue that our study has demonstrated a potential for positive outcomes for communication and psychosocial well-being following LSVT LOUD® for this group. This now needs to be investigated with larger trials to establish whether similar results could be achieved with less intensive interventions such as traditional phonatory treatment, and whether other approaches would be more effective in also addressing intelligibility and naturalness of speech in people with ataxia. However, whilst we await the outcomes of larger randomised controlled trials such as [48], we would suggest that SLTs can consider providing LSVT LOUD® treatment for patient with progressive ataxias, provided that the impact of the treatment is closely monitored for improvements and adverse effects.
